# Functional examination of *MLH1*, *MSH2*, and *MSH6* intronic mutations identified in Danish colorectal cancer patients

**DOI:** 10.1186/1471-2350-14-103

**Published:** 2013-10-03

**Authors:** Sanne M Petersen, Mette Dandanell, Lene J Rasmussen, Anne-Marie Gerdes, Lotte N Krogh, Inge Bernstein, Henrik Okkels, Friedrik Wikman, Finn C Nielsen, Thomas v O Hansen

**Affiliations:** 1 Center for Genomic Medicine, Rigshospitalet, University of Copenhagen, Blegdamsvej 9, DK-2100 Copenhagen, Denmark; 2 Center for Healthy Aging, Department of Cellular and Molecular Medicine, University of Copenhagen, Copenhagen, Denmark; 3 Department of Clinical Genetics, Rigshospitalet, University of Copenhagen, Copenhagen, Denmark; 4 Department of Clinical Genetics, Odense University Hospital, Odense, Denmark; 5 The Danish HNPCC register, Department of Gastroenterology and Clinical Research, University of Copenhagen, Hvidovre, Denmark; 6 Section of Molecular Diagnostics, Department of Clinical Biochemistry, Aalborg University Hospital, Aalborg, Denmark; 7 Department of Molecular Medicine, Aarhus University Hospital, Aarhus, Denmark

**Keywords:** Colorectal cancer, HNPCC, Lynch syndrome, Mini-gene assay, Mismatch repair genes *MLH1*, *MSH2*, and *MSH6*, Splicing defect

## Abstract

**Background:**

Germ-line mutations in the DNA mismatch repair genes *MLH1*, *MSH2*, and *MSH6* predispose to the development of colorectal cancer (Lynch syndrome or hereditary nonpolyposis colorectal cancer). These mutations include disease-causing frame-shift, nonsense, and splicing mutations as well as large genomic rearrangements. However, a large number of mutations, including missense, silent, and intronic variants, are classified as variants of unknown clinical significance.

**Methods:**

Intronic *MLH1*, *MSH2*, or *MSH6* variants were investigated using *in silico* prediction tools and mini-gene assay to asses the effect on splicing.

**Results:**

We describe *in silico* and *in vitro* characterization of nine intronic *MLH1*, *MSH2*, or *MSH6* mutations identified in Danish colorectal cancer patients, of which four mutations are novel. The analysis revealed aberrant splicing of five mutations (*MLH1* c.588 + 5G > A, *MLH1* c.677 + 3A > T, *MLH1* c.1732-2A > T, *MSH2* c.1276 + 1G > T, and *MSH2* c.1662-2A > C), while four mutations had no effect on splicing compared to wild type (*MLH1* c.117-34A > T, *MLH1* c.1039-8 T > A, *MSH2* c.2459-18delT, and *MSH6* c.3439-16C > T).

**Conclusions:**

In conclusion, we classify five *MLH1/MSH2* mutations as pathogenic, whereas four *MLH1/MSH2/MSH6* mutations are classified as neutral. This study supports the notion that *in silico* prediction tools and mini-gene assays are important for the classification of intronic variants, and thereby crucial for the genetic counseling of patients and their family members.

## Background

Lynch syndrome, also called hereditary nonpolyposis colorectal cancer (HNPCC), is an autosomal dominantly inherited cancer predisposition syndrome primarily associated with germ-line mutations in the *MLH1* (MIM# 120436), *MSH2* (MIM# 609309), and *MSH6* (MIM# 600678) genes [1]. Mutation carriers have an increased risk of several specific cancers, in particular colorectal, endometrial, small bowel, and ovarian cancer as well as uroepithelial tumors. The estimated lifetime risk of developing colorectal cancer with a pathogenic mutation in one of these genes is up to 70% [2], depending on the mutated mismatch repair gene and the gender of the patient.

The MLH1, MSH2, and MSH6 proteins are involved in the repair of single base mismatches and short insertion-deletion loops that arise during DNA replication [3]. Mutations in *MLH1*, *MSH2*, and *MSH6* are scattered throughout the genes (http://chromium.liacs.nl/LOVD2/colon_cancer/) and include frame-shift, nonsense, missense, and splice site mutations as well as large genomic rearrangements, of which several have been identified in Danish Lynch syndrome families [4-7]. However, a large number of *MLH1*, *MSH2*, and *MSH6* missense, silent, and intronic mutations are of unknown clinical significance. It is clinically important to optimize the classification of these mutations into pathogenic mutations or benign polymorphisms in order to provide affected families with a more accurate risk assessment but also to offer predictive (presymptomatic) genetic testing to family members. The classification can be facilitated by performing functional assays (reviewed by [8]). In this study, we performed *in silico* analysis and functional examinations of nine intronic *MLH1*, *MSH2*, and *MSH6* variants identified in Danish colorectal cancer patients enabling us to classify five mutations as pathogenic and four variants as neutral/polymorphisms.

## Methods

### Patients and clinical data

Following verbal and written consent blood samples were collected from the probands (all adults) and genetic screening was performed. Since the study is part of normal diagnostic procedures no ethical approval was obtained (H-4-2013-FSP-082). Clinical data regarding family phenotype, individual phenotype, cancer diagnosis, age at onset, adenomas, and age at adenomas (See Additional file 1) were obtained from the Danish HNPCC register. The study was conducted in accordance with the Helsinki Declaration.

### *MLH1*, *MSH2*, and *MSH6* screening

Genomic DNA was purified from whole blood using Qiagen’s QIAamp DNA mini kit or Promega’s Maxwell DNA purification system according to the accompanying instructions. *MLH1*, *MSH2*, and *MSH6* were amplified using intronic primer pairs flanking each exon. PCR products were sequenced using an ABI3730 DNA analyzer (Applied Biosystems). Moreover, genomic DNA was examined by MLPA analysis using kit P003 and P072 (MRC-Holland). Sequence variations, except well-known polymorphisms, were verified in a new blood sample. *MLH1*, *MSH2*, and *MSH6* variants are numbered according to GenBank accession numbers NM_000249, NM_000251, and NM_000179, respectively. The nomenclature guidelines of the Human Genome Variation Society (http://www.hgvs.org/mutnomen) were used in all cases.

### *In silico* analysis

The following five splice site prediction programs were used to predict the effect of mutations on the efficiency of splicing: Splice Site Finder (http://www.interactive-biosoftware.com); GeneSplicer (http://www.cbcb.umd.edu/software/GeneSplicer); Splice Site Prediction by Neural Network (http://www.fruitfly.org/seq_tools/splice.html); MaxEntScan (http://genes.mit.edu/burgelab/maxent/Xmaxentscan_scoreseq.html); and Human Splicing Finder (http://www.umd.be/HSF/). The analysis was performed by the integrated software Alamut V.2.2.1 (http://www.interactive-biosoftware.com). The genomic sequence spanning the individual mutations and nearby exons was submitted according to the guidelines of each program and default settings were used in all predictions. A variation of more than 10% in at least two algorithms was considered as having an effect on splicing [9].

### Mini-gene assay

Wild type exons along with at least 200 bp of 5′ and 3′ intronic sequences from *MLH1*, *MSH2*, and *MSH6* were PCR amplified from human genomic DNA using Pwo DNA polymerase (Roche) and forward and reverse primers carrying restriction sites for *Bam*HI or *Xho*I (primer sequences are available on request). PCR products were subcloned into the pSPL3 vector and all constructs were verified by sequencing. Single nucleotide substitutions or deletions were introduced using Finnzymes’ Phusion site-directed mutagenesis kit or Stratagene’s QuikChange II site-directed mutagenesis kit with PfuUltra high-fidelity DNA polymerase according to the accompanying instructions. Wild type and mutant constructs were transfected in duplicate into COS-7 cells as recently described [10]. Cells were harvested after 48 hours and total RNA was extracted using NucleoSpin RNA/protein kits for total RNA and protein isolation (Macherey-Nagel). cDNA was synthesized using 1 μg/μl of RNA, M-MuLV reverse transcriptase polymerase (New England Biolabs), and 0.5 μg/μl of nucleotide oligo(dT)_15_ primer. cDNA was amplified with Pwo DNA polymerase using the primers dUSD2 (5′-TCTGAGTCACCTGGACAACC-3′) and dUSA4 (5′-ATCTCAGTGGTATTTGTGAGC-3′). PCR products were separated by electrophoresis on a 1% agarose gel containing ethidium bromide. Each DNA band was gel purified using GE Healthcare’s Illustra GFX PCR DNA and gel band purification kit and sequenced with dUSD2 and dUSA4 primers.

## Results

Since 1995, our department has conducted screening of the entire coding regions and the exon-intron boundaries of *MLH1* and *MSH2*. Furthermore, since 2004, screening of *MSH6* and MLPA analysis of all three genes, have also been performed. During this period, a relatively broad spectrum of disease-causing germ-line *MLH1*, *MSH2*, and *MSH6* mutations has been identified [4-7]. However, mutational screening has also identified numerous variants of unknown clinical significance, including several intronic variants. Five of these intronic variants were identified in *MLH1* (c.117-34A > T; c.588 + 5G > A; c.677 + 3A > T; c.1039-8 T > A; c.1732-2A > T), three in *MSH2* (c.1276 + 1G > T; c.1662-2A > C; c.2459-18delT), and one in *MSH6* (c.3439-16C > T) (Table 1). The *MLH1* c.117-34A > T, *MLH1* c.588 + 5G > A, *MLH1* c.677 + 3A > T, *MSH2* c.1276 + 1G > T, *MSH2* c.1662-2A > C, *MSH2* c.2459-18delT, and *MSH6* c.3439-16C > T variants were identified in 7 independent families, whereas *MLH1* c.1732-2A > T was identified in two different families and *MLH1* c.1039-8 T > A was identified in six different families. The clinical data from the probands are shown in Additional file 1 (excluding IHC and MSI data that was not available from the patients). The probands belong to Amsterdam positive families (six patients), Amsterdam-like families (six patients), moderate risk families (two patients) or non-HNPCC families (one patient).

**Table 1:** ***In silico *prediction of the effect of mutations on splice donor (SD) or splice acceptor (SA) sites**

**Gene**	**IVS**	**Mutation**	**SSF (0–100)**	**MES (0–16)**	**NNS (0–1)**	**GS (0–15)**	**HSF (0–100)**
*MLH1*	1	c.117-34A > T	SA:80.80/80.80	SA:7.22/7.22	SA:0.86/0.86	SA:NI/NI	SA:84.10/84.10
	7	c.588 + 5G > A	SD:87.56/75.41	SD:9.72/4.06	SD:0.97/NI	SD:0.93/NI	SD:88.47/76.30
			(-13.9%)	(-58.2%)	(-100%)	(-100%)	(-13.8%)
	8	c.677 + 3A > T	SD:76.78/NI	SD:9.22/3.55	SD:0.98/NI	SD:NI/NI	SD:84.99/73.21
			(-100%)	(-61.5%)	(-100%)		(-13.9%)
	11	c.1039-8 T > A	SA:95.48/90.38	SA:7.50/6.74	SA:0.96/0.79	SA:7.81/5.23	SA:88.87/86.88
			(-5.3%)	(-10.1%)	(-17.7%)	(-33.0%)	(-2.2%)
	15	c.1732-2A > T	SA:86.24/NI	SA:9.34/NI	SA:0.99/NI	SA:10.34/NI	SA:84.57/NI
			(-100%)	(-100%)	(-100%)	(-100%)	(-100%)
*MSH2*	7	c.1276 + 1G > T	SD:81.67/NI	SD:8.92/NI	SD:0.91/NI	SD:0.81/NI	SD:84.70/NI
			(-100%)	(-100%)	(-100%)	(-100%)	(-100%)
	10	c.1662-2A > C	SA:85.11/NI	SA:8.01/NI	SA:NI/NI	SA:NI/NI	SA:86.96/NI
			(-100%)	(-100%)			(-100%)
	14	c.2459-18delT	SA:81.50/81.50	SA:9.97/8.33	SA:0.95/0.97	SA:5.06/4.05	SA:83.23/83.23
				(-16.4%)	(+2.1%)	(-20.0%)	
*MSH6*	5	c.3439-16C > T	SA:85.93/85.93	SA:10.55/10.32	SA:0.95/0.96	SA:8.75/9.12	SA:89.74/89.74
				(-2.2%)	(+1.1%)	(+4.2%)	

Five prediction programs were used: Splice Site Finder (SSF), MaxEntScan (MES), NNsplice (NNS), GeneSplicer (GS), and Human Splicing Finder (HSF). The thresholds represent score predicted for wt sequence/score predicted for mutated sequence. Scores indicate the values for SD or SA sites, respectively. Changes relative to wild type sequences are indicated in % (bold if >10%). IVS = intron; NI = not identified.

**Figure 1:**
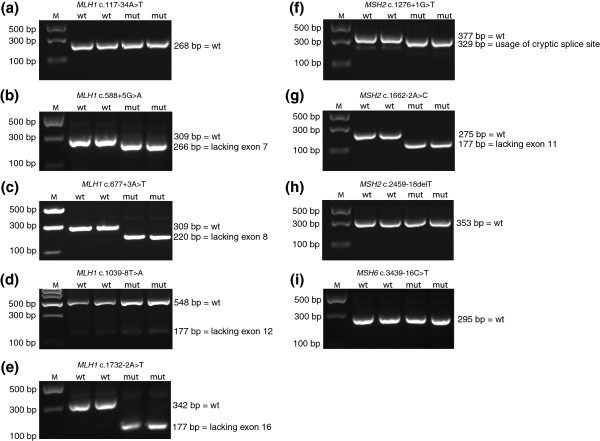
**Mini-gene analysis of *MLH1*, *MSH2*, and *MSH6 *intronic mutations.** COS-7 cells were transfected with wild type or mutant plasmids in duplicate. Total RNA was isolated, and RT-PCR analysis was performed. PCR products were separated by agarose gel electrophoresis and visualized by ethidium bromide staining. The sizes of the DNA marker (M) are indicated to the left. All PCR products were verified by sequencing. **(a)** The *MLH1* c.117-34A > T mutation produced a 268-bp PCR product which corresponds to wild type exon 2 (unaltered splicing). **(b)** The *MLH1* c.588 + 5G > A mutation produced a 266-bp band corresponding to the exclusion of exon 7. **(c)** The *MLH1* c.677 + 3A > T mutation resulted in a 220-bp band corresponding to a transcript lacking exon 8. **(d)** The *MLH1* c.1039-8 T > A mutation produced a 548-bp PCR product which corresponds to wild type exon 12 (unaltered splicing). **(e)** The *MLH1* c.1732-2A > T mutation resulted in a 177-bp transcript corresponding to the exclusion of exon 16. **(f)** The *MSH2* c.1276 + 1G > T mutation produced a 329-bp transcript by usage of a cryptic splice donor site 48 bp within exon 7. **(g)** The *MSH2* c.1662-2A > C mutation resulted in a 177-bp product corresponding to a transcript lacking exon 11. **(h)** The *MSH2* c.2459-18delT mutation produced a 353-bp PCR product which corresponds to wild type exon 14 (unaltered splicing). **(i)** The *MSH6* c.3439-16C > T mutation produced a 295-bp PCR product which corresponds to wild type exon 5 (unaltered splicing).

The potential pathogenicity of the variants was investigated using five different *in silico* splice site prediction programs which predict changes in splice site strength. The threshold employed was a variation between the wild type and the mutation score of more than 10% in at least two different algorithms [9]. According to this criterion, seven mutations, namely, *MLH1* c.588 + 5G > A, *MLH1* c.677 + 3A > T, *MLH1* c.1039-8 T > A, *MLH1* c.1732-2A > T, *MSH2* c.1276 + 1G > T, *MSH2* c.1662-2A > C, and *MSH2* c.2459-18delT (Table 1) were suggested to have an effect on splicing, whereas no splicing alterations were predicted for the two remaining mutations (*MLH1* c.117-34A > T and *MSH6* c.3439-16C > T). To verify the *in silico*-predicted effects, functional mini-gene experiments were performed on all nine intronic variants, since no RNA was available from the patients. Wild type and mutant cDNA fragments including the exon of interest and at least 200 bp of upstream and downstream intronic sequences were cloned into the pSPL3 vector and subsequently transfected into COS-7 cells (in duplicate). After 48 hours, mRNA was purified, analyzed by RT-PCR, and then visualized on 1% agarose gels (Figure 1a-i). No band size differences were observed between wild type and mutant *MLH1* c.117-34A > T (Figure 1a), *MLH1* c.1039-8 T > A (Figure 1d), *MSH2* c.2459-18delT (Figure 1h), and *MSH6* c.3439-16C > T (Figure 1i). These findings were verified by sequencing of the gel bands. In contrast, *MLH1* c.588 + 5G > A, *MLH1* c.677 + 3A > T, *MLH1* c.1732-2A > T, *MSH2* c.1276 + 1G > T, and *MSH2* c.1662-2A > C mutants all revealed the presence of alternative gel bands compared to the corresponding wild types. The wild type *MLH1* exon 7-exon 8 construct generated one transcript comprising the expected 309 bp, while both *MLH1* c.588 + 5G > A and *MLH1* c.677 + 3A > T yielded one strong band of 266 bp and 220 bp, respectively, lacking either exon 7 or exon 8 (Figure 1b and c). Wild type *MLH1* exon 16 revealed the presence of one band at the expected size of 342 bp, while *MLH1* c.1732-2A > T resulted in one strong band of 177 bp lacking exon 16 (Figure 1e). Wild type *MSH2* exon 7 exhibited one band with the expected size of 377 bp containing exon 7, while *MSH2* c.1276 + 1G > T resulted in a 329-bp transcript lacking the last 48 bp of exon 7 (Figure 1f). Moreover, wild type *MSH2* exon 11 revealed the presence of a 275-bp transcript containing exon 11, while *MSH2* c.1662-2A > C revealed a single band of 177 bp lacking exon 11 (Figure 1g).

## Discussion

Mutations located in the introns of mismatch repair genes can interfere with splicing and cause aberrant spliced mRNA transcripts leading to non-functional mismatch repair proteins. Several *cis*-acting elements, including the donor splice site, the acceptor splice site, the branch point, the polypyrimidine tract, and exonic/intronic splicing enhancers and silencers, are crucial for the splicing mechanism. The donor splice site consists of the conserved dinucleotides GT, whereas the acceptor splice site consists of three regions: the conserved dinucleotides AG, the polypyrimidine tract, and the branch point [11]. Mutations in splicing motifs can lead to partial or complete skipping of the neighboring exon or inclusion of intronic sequence. Moreover, a mutation can create an ectopic splice site or activate a cryptic splice site, both of which are usually weak and only used when a mutation disrupts the normal splice site.

Ideally RNA from a patient should be examined by RT-PCR analysis to establish if a mutation has an effect on splicing. However, in many cases, RNA is not available from the patient. Alternatively, the mutation can be examined by mini-gene analysis [12]. In fact, a high concordance between RT-PCR analysis and mini-gene assay has previously been observed [9,13-15]. As an indicative examination prior to the mini-gene assay, several *in silico* prediction tools can be used to indicate which variants require further analysis.

In this study, we examined the effect on splicing of nine intronic variants identified in Danish colorectal cancer families by *in silico* analysis and *in vitro* using a mini-gene assay. The *in silico* analysis predicted altered splicing for *MLH1* c.588 + 5G > A, *MLH1* c.677 + 3A > T, *MLH1* c.1039-8 T > A, *MLH1* c.1732-2A > T, *MSH2* c.1276 + 1G > T, *MSH2* c.1662-2A > C, and *MSH2* c.2459-18delT, whereas *MLH1* c.117-34A > T and *MSH6* c.3439-16C > T were predicted to have no effect on splicing. It should be noted that three mutations in our study (*MLH1* c.1732-2A > T, *MSH2* c.1276 + 1G > T, and *MSH2* c.1662-2A > C) are located in the highly conserved donor and acceptor splice sites and hence they are easily predicted by *in silico* programs. However, mini-gene analysis revealed that the two mutations *MLH1* c.1039-8 T > A and *MSH2* c.2459-18delT had no effect on splicing, suggesting that the employed criterion (>10% difference between wild type and mutant scores in at least two programs) results in false-positive predictions as previously shown [9].

Mini-gene analysis revealed that the *MLH1* c.117-34A > T and *MLH1* c.1039-8 T > A variants had no effect on splicing. The *MLH1* c.117-34A > T variant has not been described before, whereas our results regarding *MLH1* c.1039-8 T > A confirm previous data analyzing patient RNA [16]. Moreover, in one Amsterdam positive family (H13), the *MLH1* c.1039-8 T > A mutation was identified together with a disease-causing *MLH1* mutation (c.1276C > T, p.Gln426X). In conclusion we classify both variants as neutral (Table 2). In contrast, the *MLH1* c.588 + 5G > A and *MLH1* c.677 + 3A > T mutations were found to lead to exclusion of exon 7 and exon 8, respectively. Ultimately, this leads to premature stop codons and, therefore, both mutations are classified as disease-causing. These findings confirm previous results showing that the *MLH1* c.588 + 5G > A mutation causes either partial skipping/deletion of exon 7 examining patient RNA [17] or skipping of exon 7 as well as both exons 7 and 8 assesed using mini-gene assay [15], and by results from *MLH1* c.677 + 3A > C and *MLH1* c.677 + 3A > G mutations showing skipping of exon 8 [18,19]. Moreover, our analysis found that *MLH1* c.1732-2A > T, which is a Danish founder mutation identified in two Amsterdam positive families (See Additional file 1), results in an in-frame deletion of exon 16, which contains the PMS2 interaction domain. In the 2 families the mutation co-segregates with the disease and has a lod score of 1.2 and 2.7, respectively, and in agreement with previous reports [5,7,20] we therefore classify *MLH1* c.1732-2A > T as pathogenic. The *MSH2* c.1276 + 1G > T mutation was found to result in the activation of a cryptic splice donor site 48 bp within exon 7, leading to an in-frame deletion of 16 amino acids in the MSH6/MSH3 interaction domain. As the mutation was found to co-segregate in the affected family with a lod score of 1.5, it is regarded as pathogenic. This mutation has previously been described in microsatellite instability-high colorectal cancers, with immunohistochemical analysis of these tumors revealing the absence of nucleic MSH2 expression [21]. Similar results and conclusions have been reported for the *MSH2* c.1276 + 1G > A mutation [16]. The *MSH2* c.1662-2A > C mutation has not previously been described. We found that this mutation leads to skipping of exon 11 and consequently introduce a premature stop codon. Therefore, this mutation is classified as pathogenic. Finally, the *MSH2* c.2459-18delT and *MSH6* c.3439-16C > T variants were found to have no effect on splicing and are, therefore, classified as neutral. The *MSH2* c.2459-18delT variant has not been described before, whereas the *MSH6* c.3439-16C > T variant has previously been shown not to co-segregate with the disease and to be observed in healthy control individuals [22-24] and in the exome sequencing project (ESP) database (0.43%), thereby supporting the notion that this variant is neutral.

**Table 2:** The effect on splicing determined by mini-gene assays and an overview of the mutations listed in the literature

**Gene**	**IVS**	**Mutation**	**Frequency in the ESP database (Eur. Am.)**	**Mini-gene assay**	**Described in the literature**	**Classification**
*MLH1*	1	c.117-34A > T	NI	No effect on splicing	Novel	Neutral
	7	c.588 + 5G > A	NI	Out-of-frame skipping of exon 7	Pagenstecher; partial deletion of exon 7 [17]	Pathogenic
					Tournier; deletion of exon 7 and exons 7–8 [15]	
	8	c.677 + 3A > T	NI	Out-of-frame skipping of exon 8	Novel	Pathogenic
	11	c.1039-8 T > A	NI	No effect on splicing	Betz; No effect on splicing [16]	Neutral
	15	c.1732-2A > T	NI	In-frame skipping of exon 16	Jäger* [5]	Pathogenic
					Nilbert* [7]	
					Wijnen* [20]	
*MSH2*	7	c.1276 + 1G > T	NI	In-frame exclusion of 48 bp of exon 7	Mangold* [21]	Pathogenic
	10	c.1662-2A > C	NI	Out-of-frame skipping of exon 11	Novel	Pathogenic
	14	c.2459-18delT	NI	No effect on splicing	Novel	Neutral
*MSH6*	5	c.3439-16C > T	0.43%	No effect on splicing	Perez-Cabornero* [22]	Neutral
					Pinto* [23]	
					Sanchez de Abajo* [24]	

*The authors describe the mutation of interest, but do not examine its putative pathogenic effect. IVS = intron; NI = not identified.

Overall, in all Amsterdam positive families - except one (H229) - a pathogenic mutation was identified. The index individual in family H229 had rectum cancer at age 58 and transverse colon cancer at age 66. His sister and two maternal cousins all had adenomas, while his mother has caecum cancer at age 48. Moreover his maternal aunt had transverse colon cancer at age 69 and his maternal grandmother had ascending colon cancer. The lack of a pathogenic mutation in this family could be due to an unidentified mutation in regions not previously examined, including the promoter region, the untranslated regions (UTRs) or deep intron sequences in the *MLH1*, *MSH2* or *MSH6* genes, or due to a mutation in other genes like *PMS2*. Future studies using exome sequencing might help identifying a putative pathogenic mutation in this family.

## Conclusion

In conclusion, we have examined nine *MLH1*/*MSH2*/*MSH6* intronic mutations by *in silico* and functional assays, thus enabling us to classify five mutations as pathogenic and four variants as neutral/polymorphisms. This study supports the notion that *in silico* prediction tools and mini-gene assays are important for the assessment of the pathogenicity of intronic variants, together with clinical data, IHC and MSI.

## Competing interests

The authors declare that they have no competing interests.

## Authors’ contributions

TvOH designed the study. AMG, LNK and IB were involved in recruitment of patients and the collection of clinical data, while FCN, TvOH, HO and FW were involved in the genetic screening of the patients. TvOH and SMP performed the *in silico* analysis, and SMP and MD performed the mini-gene analysis. TvOH and SMP drafted the manuscript, while MD, LJR, AMG, LNK, IB, HO, FW, and FCN were involved in the revision of the manuscript. All authors read and approved the final version of the manuscript.

## Pre-publication history

The pre-publication history for this paper can be accessed here:

http://www.biomedcentral.com/1471-2350/14/103/prepub

## Supplementary Material

Additional file 1 **Clinical data from individuals with *MLH1*, *MSH2*, *MSH6 *intron mutations.** Click here for file
