# Supplementary material for: Functional examination of MLH1, MSH2, and MSH6 intronic mutations identified in Danish colorectal cancer patients
Source: BMC Med Genet. 2013 Oct 3;14:103. doi: 10.1186/1471-2350-14-103 (PMC3850734; doi:10.1186/1471-2350-14-103)
Supplement: Additional file 1 — Clinical data from individuals with MLH1, MSH2, MSH6 intron mutations. [file 1471-2350-14-103-S1.docx]

| **Gene** | **Mutation** | **Personal ID** | **Phenotype-family** | **Co-Segregation** | **Lod score** | **Person analyzed** | **Cancer diagnosis** | **Age cancer** | **Adenomas** | **Age**  **adenoma** |
| --- | --- | --- | --- | --- | --- | --- | --- | --- | --- | --- |
| *MLH1* | c.117-34A>T | H605 | **Amsterdam-like**  (2pt with CRC and 1pt with pancreatic cancer) | ND |  | Affected | Sigmoid colon  Caecum | 47  47 |  |  |
| *MLH1* | c.588+5G>A | H14 | **Amsterdam Pos** | Yes | 1.5 | Affected | Transverse colon  Sigmoid colon  Malingnant melanoma  Kerathoacantom | 54  54  58  58 | 1 | 54 |
| *MLH1* | c.677+3A>T | H4078 | **Moderate Risk**  (1pt with CRC < age 50 yrs) | ND |  | Affected | Sigmoid | 18 |  |  |
| *MLH1* | c.1039-8T>A | H229 | **Amsterdam Pos** | ND |  | Affected | Rectum  Transverse colon | 58  66 | 2 | 64, 66 |
| *MLH1* | c.1039-8T>A | H1000 | **Amsterdam-like**  (2pt with CRC and 1pt with kidney cancer) | ND |  | Affected | Caecum | 49 |  |  |
| *MLH1* | c.1039-8T>A | H422 | **Moderate Risk**  (2pt with CRC and 2pt with adenoma, large family with skipped generation) | ND |  | Unaffected  (pt with adenoma) |  |  | 2 | 49, 55 |
| *MLH1* | c.1039-8T>A* | H13 | **Amsterdam Pos** | ND |  | Affected | Descending colon |  |  |  |
| *MLH1* | c.1039-8T>A | H315 | **Amsterdam-like**  (4pt with CRC in the  same generation) | ND |  | Affected | Colon unspecified |  |  |  |
| *MLH1* | c.1039-8T>A | H340 | **Amsterdam-like**  (1pt with CRC, 1pt CRC + metachron endometrial cancer and 1pt adenoma) | ND |  | Affected | Sigmoid colon | 49 | 3 | 55, 58, 64 |
| *MLH1* | c.1732-2A>T | H3 | **Amsterdam Pos** | Yes | 1.2 | Affected | Endometrial | 52 | 6 | 44, 45, 48, 53 |
| *MLH1* | c.1732-2A>T | H5 | **Amsterdam Pos** | Yes | 2.7 | Affected | Caecum  Sigmoid colon  Sigmoid colon  Jejunum  Skin (squamous cell)  Prostate | 47  52  55  56  72  72 |  |  |
| *MSH2* | c.1276+1G>T | H11 | **Amsterdam Pos** | Yes | 1.5 | Affected | Rectum  Hepatic flexure  Ascending colon  Kidney | 42  63  63  66 |  |  |
| *MSH2* | c.1662-2A>C | H495 | **Amsterdam-like**  (3pt CRC. Skipped generations) | Yes | 1.2  (two healthy obligate carriers) | Affected | Transverse colon  Splenic flexure | 20  28 |  |  |
| *MSH2* | c.2459-18delT | H380 | **Amsterdam-like**  (3pt with CRC, Skipped generation through 1pt with glioblastoma) | ND |  | Affected | Rectum | 45 |  |  |
| *MSH6* | c.3439-16C>T | H1233 | **Not HNPCC**  (1pt with CRC, 1pt ovarian cancer and 1pt with four carcinoid-tumours in jejunum) | ND |  | Affected | Ascending colon | 65 |  |  |

**Additional file 1 Clinical data from individuals with *MLH1*, *MSH2*, *MSH6* intron mutations**

Abbreviation: Amsterdam Pos, Amsterdam positive; CC, colon cancer ; CRC, colorectal cancer; ND, not done; Pt, patient/patients; RC, rectal cancer. Amsterdam positive family is defined as a family with at least 3 individual with verified CRC (histology, medical record or death-certificate), in two generation, one is first degree relatives to the others, and where Familial Adenomatous Polyposis is excluded. Amsterdam-like families look like Amsterdam positive family, but the Amsterdam criteria are not fulfilled. The phenotype of the pedigree is explained in brackets. Moderate Risk is defined as aggregation of CRC in the family. The phenotype of the pedigree explained in brackets. * Pathogenic mutation (*MLH1* c.1276C>T, p.Gln426X) is identified in this family.
